# Nitenpyram biodegradation by a novel nitenpyram-degrading bacterium, *Ochrobactrum* sp. strain DF-1, and its novel degradation pathway

**DOI:** 10.3389/fmicb.2023.1209322

**Published:** 2023-07-13

**Authors:** Guangli Wang, Mengqing Chen, Li Jiang, Yunfang Zhang

**Affiliations:** ^1^Anhui Province Key Laboratory of Pollution Sensitive Materials and Environmental Remediation, School of Life Sciences, Huaibei Normal University, Huaibei, China; ^2^State Key Laboratory of Desert and Oasis Ecology, Xinjiang Institute of Ecology and Geography, Chinese Academy of Sciences, Urumqi, China

**Keywords:** *Ochrobactrum* sp. DF-1, biodegradation, nitenpyram, metabolic pathway, bioremediation

## Abstract

Nitenpyram is a neonicotinoid insecticide that is commonly found in the environment. However, its biodegradation by pure cultures of bacteria has not been widely investigated and the catabolic pathway (s) for nitenpyram metabolism remain elusive. In this study, the aerobic strain DF-1, isolated from a wastewater-treatment pool contaminated with nitenpyram. The strain was designated an *Ochrobactrum* sp. utilizing a combination of traditional methods and molecular ones. Strain DF-1 can use nitenpyram as a sole carbon or nitrogen source for growth. In liquid medium, 100 mg·L^−1^ nitenpyram was metabolized to undetectable levels within 10 days. Four metabolites were found by gas chromatography–mass spectrometry (GC–MS) analyses during nitenpyram degradation. According to the aforementioned data, a partial metabolic pathway of nitenpyram was proposed of strain DF-1. Inoculation of strain DF-1 promoted nitenpyram (10 mg·kg^−1^) degradation in either sterile or non-sterile soil. To our knowledge, this is the first characterization of nitenpyram degradation by a specific bacterium and likely to be exploited for the remediation of nitenpyram-contaminated sites.

## 1. Introduction

The neonicotinoid class of insecticides is the most efficient at controlling sucking pests, such as aphids, whiteflies, leafhoppers and planthoppers, thrips, several of the micro-lepidoptera, and some coleopteran pests. The multiple functions of these insecticides are derived from their physicochemical properties, which allow different modes of application of these insecticides, including foliar and stem application, seed treatment, and soil drench (Rouchaud et al., 1994; Nauen et al., 2003; Elbert et al., 2008). Nitenpyram, a second-generation neonicotinoid family pesticide, marketed in 1995, is characterized by its relatively low mammalian toxicity and no apparent long-term cumulative effects (Veeranagouda et al., 2006; Sun et al., 2013). However, the extensive use of nitenpyram, along with other neonicotinoids, has created a significant toxicity problem for non-target, economically vital insects (Phugare and Jadhav, 2014). Nitenpyram as one of the most frequently detected neonicotinoid insecticides has high water solubility (590 g·L^−1^) and therefore relatively short persistence in soil, resulting in its easily leaching to contaminate groundwater and surface water (Ahmad et al., 2021; Dai et al., 2021). Therefore, nitenpyram accumulation in the environment should be prevented or at least mitigated. Microbial degradation has attracted attention as an efficient and cost-efficient biotechnological strategy for removing hazardous compounds from the environment (Cabrera et al., 2010). A strategy for the remediation of neonicotinoids that includes the efficient degradation by microorganisms is urgently needed. Several microorganisms capable of degrading nitenpyram have been isolated (Wang et al., 2019; Pang et al., 2020). Several metabolites of nitenpyram due to microbial degradation have been reported (Wang et al., 2019; Pang et al., 2020). Until now, there was no clear biochemical degradation pathway of nitenpyram reported in a microorganism.

We here isolated and characterized an *Ochrobactrum* strain, designated DF-1, that can grow by utilizing nitenpyram as a sole carbon or nitrogen source. A few parameters, namely pH, temperature, and initial substrate concentration, were examined to determine their effects on nitenpyram degradation by the isolated strain. Moreover, a pathway of strain DF-1-mediated nitenpyram degradation was suggested. We also reported the outcomes of a pilot-scale bioremediation experiment conducted using strain DF-1 with nitenpyram-contaminated soil. This research demonstrates a potential application of pure microbial cell cultures for nitenpyram degradation.

## 2. Materials and methods

### 2.1. Chemicals and organisms

An analytical grade (99% pure) nitenpyram (Sigma-Aldrich, Munich, Germany) was used as a reference. Nitenpyram samples (>95% purity) were purchased from China-based Nantong Jiangsu Agrochemical and Chemical Co., Ltd. A chromatographic grade methanol was purchased from Sigma-Aldrich (St. Louis, MO, United States). Furthermore, all other chemicals and reagents were of the highest commercial grade accessible. Two types of media were used in this experiment: Luria-Bertani (LB) medium and mineral salts medium (MM) were prepared according to Li et al. (2012). In practical terms, LB contained 10.0 g L^−1^ tryptone, 5.0 g·L^−1^ yeast extract, and 5.0 g L^−1^ NaCl, pH 7.0. MM contained 1.5 g·L K_2_HPO_4_, 0.5 g·L KH_2_PO_4_, 1.0 g·LNH_4_NO_3_, 0.2 g·L MgSO_4_·7H_2_O, 1.0 g·L NaCl. Nitenpyram was dissolved in acetone, sterilized by filtration. When nitenpyram was the only nitrogen source, NH_4_NO_3_ was removed from MM and glucose (1.0 g·L^−1^) was added to the liquid media. Plates with solid media were produced by adding 15 g·L^−1^ agar to the liquid media, as previously described. When necessary, nitenpyram was added at a suitable concentration to the medium. Media were sterilized at 121°C for 25 min. Ultra-pure water was used to dissolve nitenpyram, while using a 0.22-μm-pore membrane to filter out other bacteria and impurities. Thus, a nitenpyram stock solution was obtained (1.0%, w/v).

### 2.2. Strain isolation and characterization

In order to isolate nitenpyram-degrading strains, we chose to visit Nantong Jiangsu Agrochemical and Chemical Co., Ltd. (Jiangsu, China) to seek a factory that uses nitenpyram and has been established for a long time. Furthermore, we collected some activated sludge from a wastewater treatment pool in this factory. In this study, we applied a selective enrichment approach by using MM containing 50 mg·L^−1^ of nitenpyram, the only carbon and energy source, to isolate strains. Then, culture flasks were placed on a rotary shaker at 150 rpm and 30°C. After five transfers, MM agar plates supplemented with 200 mg·L^−1^ nitenpyram were incubated with serial dilutions of the cultures at 30°C for a week. Then, the colonies showing evidence of nitenpyram degradation were picked and restreaked (three times) for purity. The nitenpyram concentration was measured over 7 days through high-performance liquid chromatography (HPLC), and the degradation percentage was estimated as nitenpyram residual concentration in liquid cultures. A bacterial strain with an ability to utilize nitenpyram as a sole carbon or nitrogen source for growth, was selected for further analysis.

The novel isolate was partially described using *Bergey’s Manual of Determinative Bacteriology* (Holt et al., 1994), and 16S rRNA gene sequence analysis was performed along with examination of its morphological, physiological, and biochemical characteristics. Genomic DNA was extracted through high-salt precipitation (Sambrook and Russell, 2001). The 16S rRNA gene was amplified using a set of universal primers 27F/1492R (Wang et al., 2011). In order to improve the ligation efficiency, it is purified using a PCR purification kit (Axygen, Corning, NY, United States), then the purified PCR product was ligated into the vector pMD18-T (TaKaRa Biotechnology, Dalian, China). Subsequently, it was used to transform *Escherichia coli* DH5α. Invitrogen Biotechnology Co., Ltd. performed sequencing (Shanghai, China). Pairwise sequence similarity was determined using EzTaxon server’s global alignment method (Chun et al., 2007). After repeated alignment of the sequencing data with CLUSTAL X (Thompson et al., 1997), phylogenetic analysis was performed using MEGA, version 6.0 (Zhang et al., 2013).

G + C content in DNA was measured through thermal denaturation (Marmur and Doty, 1962) by using a Beckman DU 800 spectrophotometer (Beckman Coulter, Inc., Pasadena, CA, United States); *E. coli* K12 DNA was used as a reference.

### 2.3. Nitenpyram degradation by strain DF-1 in liquid culture

The bacterial strain DF-1 was precultured in LB and then collected at room temperature through centrifugation at 6,000 *g* for 5 min. Then, the cells were washed three times with sterilized MM. After the cell density had been adjusted to about 1.0 × 10^8^ CFU mL^−1^ (MM). Unless specifically stated, all tests were conducted with 5% (v/v) cells. The cells were then inoculated into 100 mL MM containing 100 mg·L^−1^ nitenpyram before being cultured at pH 7.0 in a rotary shaker (160 rpm, 30°C) during the experimental duration. Liquid media containing the insecticide at the same concentration and without inoculation of strain DF-1 maintained and tested in the same manner were used as control. The degradation efficiency was estimated as percentage of nitenpyram degradation of the initial concentration in the liquid culture. To identify the optimal conditions for rapid growth and nitenpyram biodegradation by DF-1, the impact of varying medium pH values (3.0, 5.0, 7.0, 9.0, and 11.0), incubation temperatures (10, 20, 30, 37, 42, and 45°C), and initial nitenpyram concentrations (50, 100, 150, 200, 250, and 300 mg·L^−1^) were investigated. Each treatment was conducted in triplicate.

### 2.4. Biodegradation of nitenpyram in soil

In this study, we collected soil samples at a depth of 0–20 cm from a vegetable garden. This garden had never been treated with nitenpyram (Huaibei, China). The samples were filtered through a 2-mm-thick sieve and stored at 4°C in the dark for 2 weeks before use. The soil’s physicochemical properties were as follows (g/kg of dry weight): organic matter, 11.2; total N, 0.6; total P, 0.5; total K, 17.8; and pH, 6.89.

The soil sample (200 g) was added to 500-mL Erlenmeyer flasks, and the content was limited to approximately 40% of its whole volume to verify the latent ability of strain DF-1 to degrade soil nitenpyram. To maintain a constant soil moisture content, we added distilled water as required throughout the experiments. The ultimate nitenpyram concentration in distilled water solution was 10 mg kg^−1^ of soil. After mixing nitenpyram in distilled water, drip irrigation was used to transfer the bacterial suspensions (in triplicate) to soils, resulting in an ultimate concentration of 1.0 × 10^8^ CFU·g^−1^ of soil, which was subsequently cultured at 30°C. Sterile conditions were used to completely mix the inoculum for inoculation. The effectiveness of nitenpyram removal from soils was compared using sterilized soil, which was prepared by autoclaving the soil twice every 2 days before use. Soils non-inoculated but treated with nitenpyram at the same concentration (in triplicates) were used as controls. At each sampling time, soil samples (10 g) were used for chemical analysis. The extraction and investigative procedures followed a similar pattern to those employed for liquid medium.

### 2.5. Analysis of chemical aspects

To determine bacterial growth, a UV–Vis recording spectrophotometer (Shimadzu Corp., Kyoto, Japan) was used to measure absorbance of bacterial cultures diluted 10 or 20 times with deionized water at 600 nm.

An equivalent volume of dichloromethane was used to extract nitenpyram from liquid cultures. After drying over anhydrous Na_2_SO_4_, a centrifugal evaporator was used to achieve evaporation by reducing the pressure at room temperature. Methanol (1 mL) was used to dissolve the residues. Subsequently, a 0.22-μm Millipore membrane (Millipore Corp., Darmstadt, Germany) was used to filter the samples in methanol. HPLC analysis was performed on all samples using separation-148 columns (4.6-mm inner diameter, 25-cm length) loaded with Kromasil 100-5C_18_. A 70: 30 v: v mixture of methanol and water was used as the mobile phase, with an elution rate of 1.0 mL·min^−1^ and an injection volume of 20 μL. A UV-900 wavelength absorbance detector used to measure column elution at 245 nm. A calibration curve was used to estimate nitenpyram concentration. Recovery efficiency of the stated method was evaluated at the concentrations of 20, 40, 60, 80 and 100 mg·L^−1^ nitenpyram that appended in MM.

To identify the metabolites produced during nitenpyram degradation, GC–MS analyses were performed on a Thermo Trace DSQ mass spectrometer, under the following conditions. Helium (purity, 99.996%) was used as a carrier gas at a flow rate of 1.2 mL min^−1^. Gas chromatography was conducted using a RTX-5 MS column (15 m × 0.25 mm × 0.25 mm, Restek Corp., United States). The column temperature was increased from 50 (1.5 min hold) to 200°C (1 min hold) at 20°C min^−1^ and then from 200 to 280°C (20 min hold) at 40°C min^−1^. The injector temperature was set at 220°C with a split ratio of 20: 1. The interface temperature and ion source temperature were both set to 280°C, and the mass was scanned in the range from 50 to 650 m/z. The column outlet was inserted directly into the electron ionization source block, operating at 70 eV. The external standard calibration method was used to measure the quantity of substrates and products.

### 2.6. Identification of nitenpyram degradation metabolites

To detect strain DF-1 metabolites, we inoculated the strain into 200 mL liquid MM media containing 200 mg·L^−1^ nitenpyram. A negative control was similarly prepared except that the inoculated cells were heatkilled. When nitenpyram degradation had reached 30, 50, and 70%, liquid culture samples were obtained for analysis. Three samples were mixed and extracted with the same proportion of dichloromethane. The possible metabolites from nitenpyram degradation were extracted twice from liquid culture with the same proportion of ethyl acetate, we used 1 N HCl to acidify the remaining aqueous phase to pH 3.0. As mentioned previously, all extracts were evaporated and the new solutions were prepared in methanol with a volume of 2 mL, and then the resultant liquid was infused into the GC–MS apparatus at a volume of 2 μL.

## 3. Results

### 3.1. Evaluation of the analytical method for nitenpyram determination

The recovery efficiency of nitenpyram in MM are arranging from 96 to 99%. The evaluation results displayed that the analytical method applied in this study well satisfied the requirement of the pesticide analysis standard (Table 1; satisfy scopes are from 80 to 120%). The coefficient of association and regression equation are shown in Table 1.

**Table 1:** Recovery efficiency and regression equation determined by different concentrations of nitenpyram for the analytical method evaluation.

Append concentration (mg·L^−1^)	Detected concentration	Recovery efficiency (%)	Regression equation
20	19.87 ± 0.53	99	
40	38.89 ± 1.45	97	y = 12.263x + 1.0012
60	57.99 ± 0.32	97	*R*^2^ = 0.9999
80	77.82 ± 2.15	97	
100	94.08 ± 2.15	96	

Regression equation was determined by HPLC using different concentrations of standard nitenpyram; y, nitenpyram concentration; x, the peak area of HPLC; R^2^, correlation coefficient of the regression equation.

### 3.2. Strain isolation and recognition

**Figure 1:**
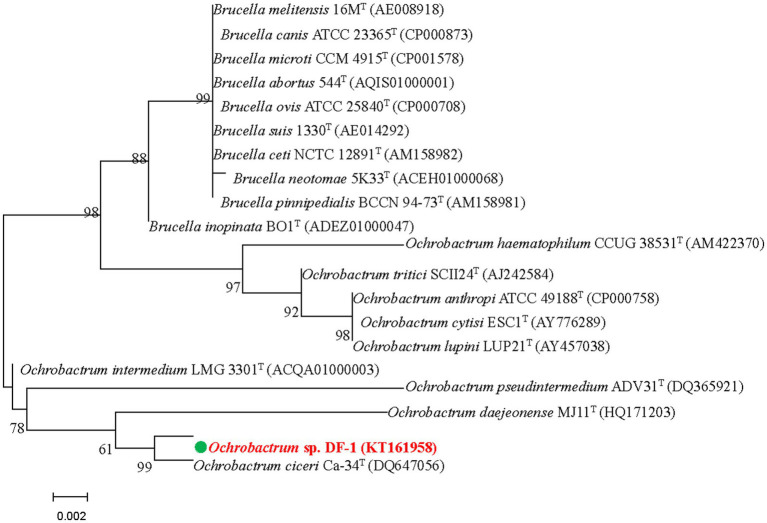
Based on the neighbor-joining method and 16S rRNA gene sequences of DF-1 and type strains of *Ochrobactrum* species, we established the phylogenetic tree. Bootstrap values, expressed as percentages of 1,000 replications, are given at branching points. Bars represent 0.005 nucleotide substitutions per nucleotide position.

The DF-1 bacterial strain was isolated from the enrichment of a wastewater pool containing nitenpyram. After 24 h of growth on LB agar at 37°C, smooth, glossy, and focally dense colonies were formed. Microscopic observation revealed that the colonies were formed by gram-negative, rod-shaped, usually circular, and low convex cells. Strain DF-1 was positive for nitrate reduction, cytochrome-*c* oxidase, and hydrogen sulfide generation, whereas negative for gelatinase, indole production, lysine and arginine dihydrolase, and ornithine decarboxylase. G + C content in DNA of the strain was 57.8 mol%. Based on these traits and other morphological, nutritional, and biochemical characteristics, strain DF-1 was provisionally recognized as a member of the genus *Ochrobactrum* (Imran et al., 2010). The 16S rRNA gene sequence of DF-1 (1,486 bp) was determined for confirmation and preserved in GenBank under the accession number KT161958. A BLAST search and comparison of that sequence with sequences available in the GenBank nucleotide database indicated a high degree of similarity to *Ochrobactrum ciceri* Ca-34^T^ (99.60%), followed by *O. intermedium* LMG 3301^T^ (98.90%). Figure 1 depicts the phylogenetic relationship of strain DF-1 with the aforementioned strains and other members of the genus *Ochrobactrum* based on 16S rRNA gene sequence similarity. A high connection was observed between the results of the combined analyses and DF-1’s biochemical characteristics at the genus level. However, when compared to the closest members of the *Ochrobactrum* genus, only a few differences were found (data not shown), which made identifying the strain to the species level difficult (Velasco et al., 1998; Imran et al., 2010). Therefore, the bacterium was recognized as *Ochrobactrum* sp. strain DF-1.

### 3.3. Biodegradation of nitenpyram by strain DF-1

**Figure 2:**
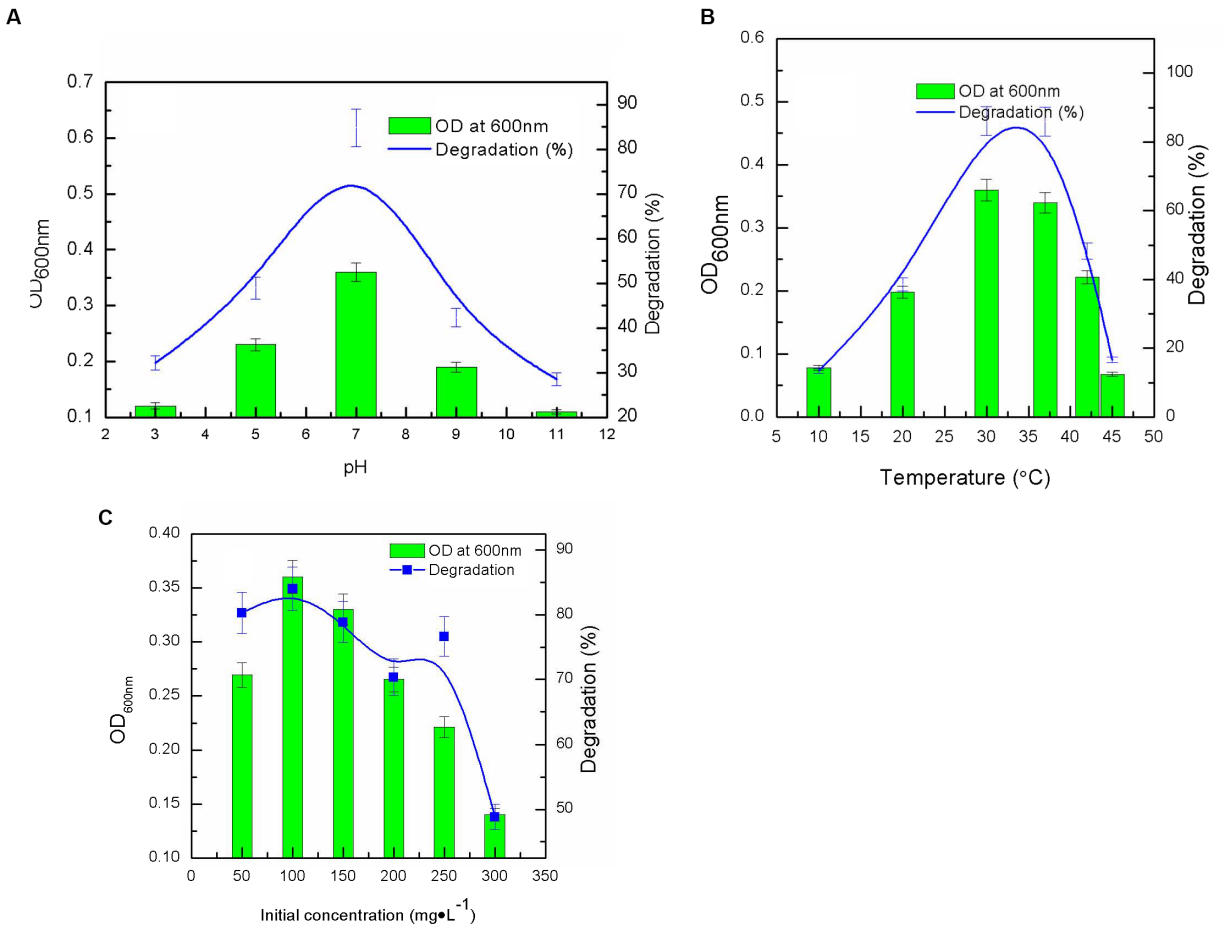
Influence of different culture conditions on strain growth (OD_600 nm_), and nitenpyram degradation by the *Ochrobactrum* sp. strain after 6 days of incubation: **(A)** pH; **(B)** temperature; **(C)** nitenpyram initial concentration. Error bars, mean ± SD of three replicates.

After a 7-day incubation period, nitenpyram biodegradation by DF-1 was assessed under various pH and temperature settings. DF-1 could degrade more than 80% of 100 mg·L^−1^ nitenpyram at pH 7.0 with a maximum OD_600nm_ of 0.360. However, at pH levels of 3.0, 5.0, 9.0, and 11.0, nitenpyram biodegradation was significantly reduced, suggesting that strain DF-1 growth was limited at these pH levels (Figure 2A). The maximum degradation and growth of strain DF-1 occurred at 30°C, while any increases or decreases in temperature from 30°C decreased biodegradation. This implies that nitenpyram catabolism in strain DF-1 is optimal in a mesophilic habitat (Figure 2B). To ascertain the influence of initial nitenpyram concentration on degradation efficiency, DF-1-mediated biodegradation was assessed at initial nitenpyram concentrations ranging from 50 to 300 mg·L^−1^. Increasing the pesticide’s concentration was detrimental for bacterial growth and slowed the biodegradation rate. Biodegradation progressed to 80.28% at 50 mg·L^−1^, whereas it decreased to 48.84% at 300 mg·L^−1^ nitenpyram. However, the maximum concentration required for nitenpyram degradation was 100 mg·L^−1^ (Figure 2B). Thus, optimal conditions (namely, pH 7.0, 30°C, and 100 mg·L^−1^) were chosen for all subsequent experiments (Figure 2).

**Figure 3:**
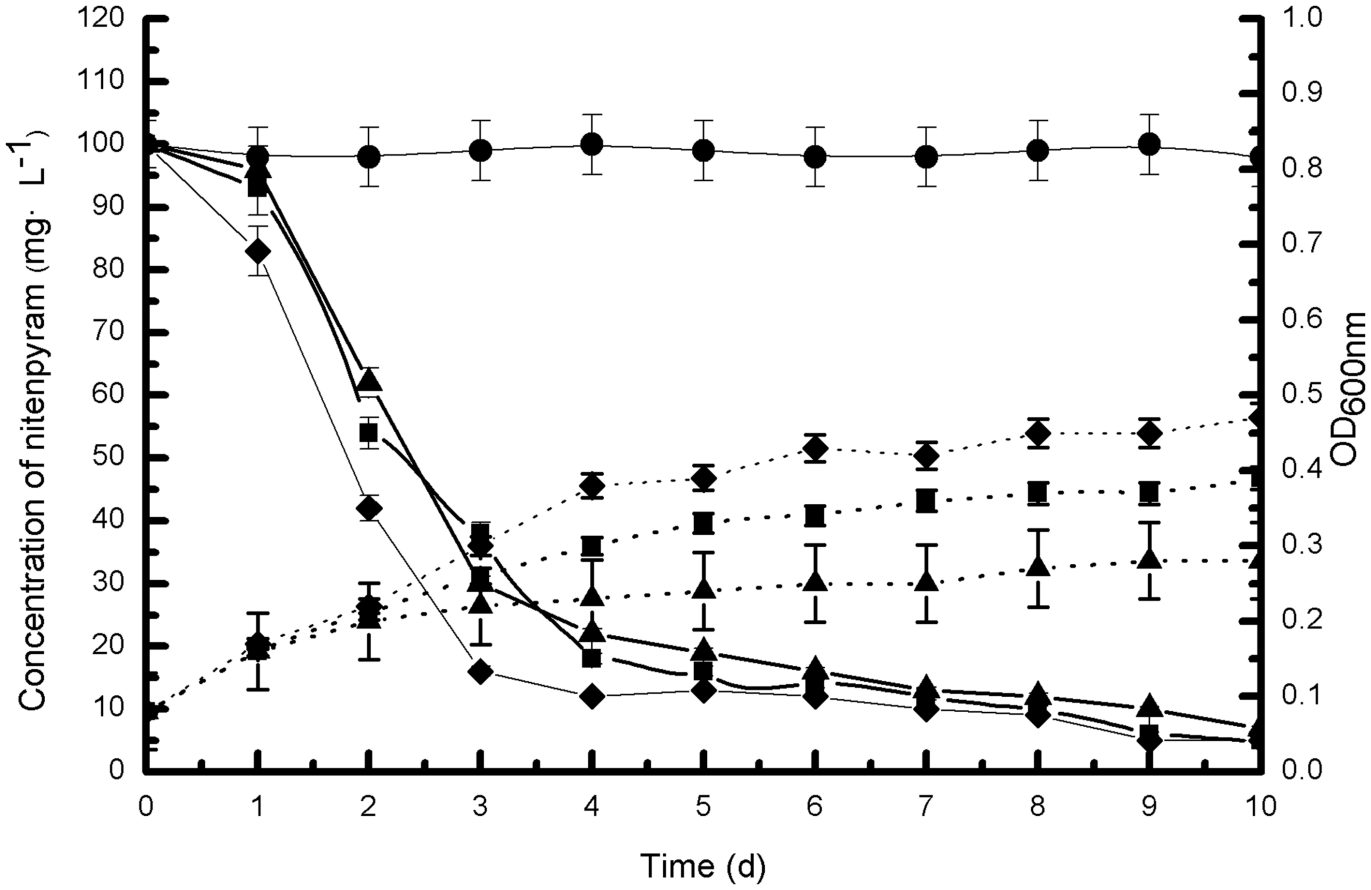
Biodegradability of strain DF-1 by utilizing nitenpyram as the only carbon or nitrogen source, and as a source of both carbon and nitrogen. OD_600 nm_ readings were used to record growth. Nitenpyram degradation by strain DF-1 is presented by solid lines, and the growth is presented by dashed lines. Nitenpyram control (●). At all instances, nitenpyram as the only carbon source (■), nitenpyram as the only nitrogen source (◆), and nitenpyram as a source of both carbon and nitrogen (▲). The standard error of three repetitions is expressed by the error bars.

Figure 3 illustrates the growth of strain DF-1 on nitenpyram and its ability to degrade nitenpyram. The OD_600 nm_ measurements revealed a consistent increase in bacterial density. At the same time, HPLC analysis revealed the nitenpyram content was significantly decreased. After 7 days of culturing with nitenpyram as the only nitrogen source, approximately 90% of the initial nitenpyram concentration of 100 mg·L^−1^ in MM medium was consumed by strain DF-1. With nitenpyram as the only carbon source, strain DF-1 required 8 days to achieve the same extent of degradation (90% of 100 mg·L^−1^ nitenpyram). However, DF-1 required 9 days when nitenpyram was used as both the only carbon and nitrogen source. The cultures not inoculated with strain DF-1 showed no change in nitenpyram concentration. Nitenpyram biodegradation was accompanied by an increase in OD_600 nm_ from approximately 0.08 to 0.39, 0.08 to 0.47, and 0.08 to 0.28 when nitenpyram was added as the only carbon source, nitrogen source, and both only carbon and nitrogen source, respectively. These data indicate that nitenpyram can serve as both carbon and nitrogen source for strain DF-1 growth.

### 3.4. Biodegradation of nitenpyram in soil

**Figure 4:**
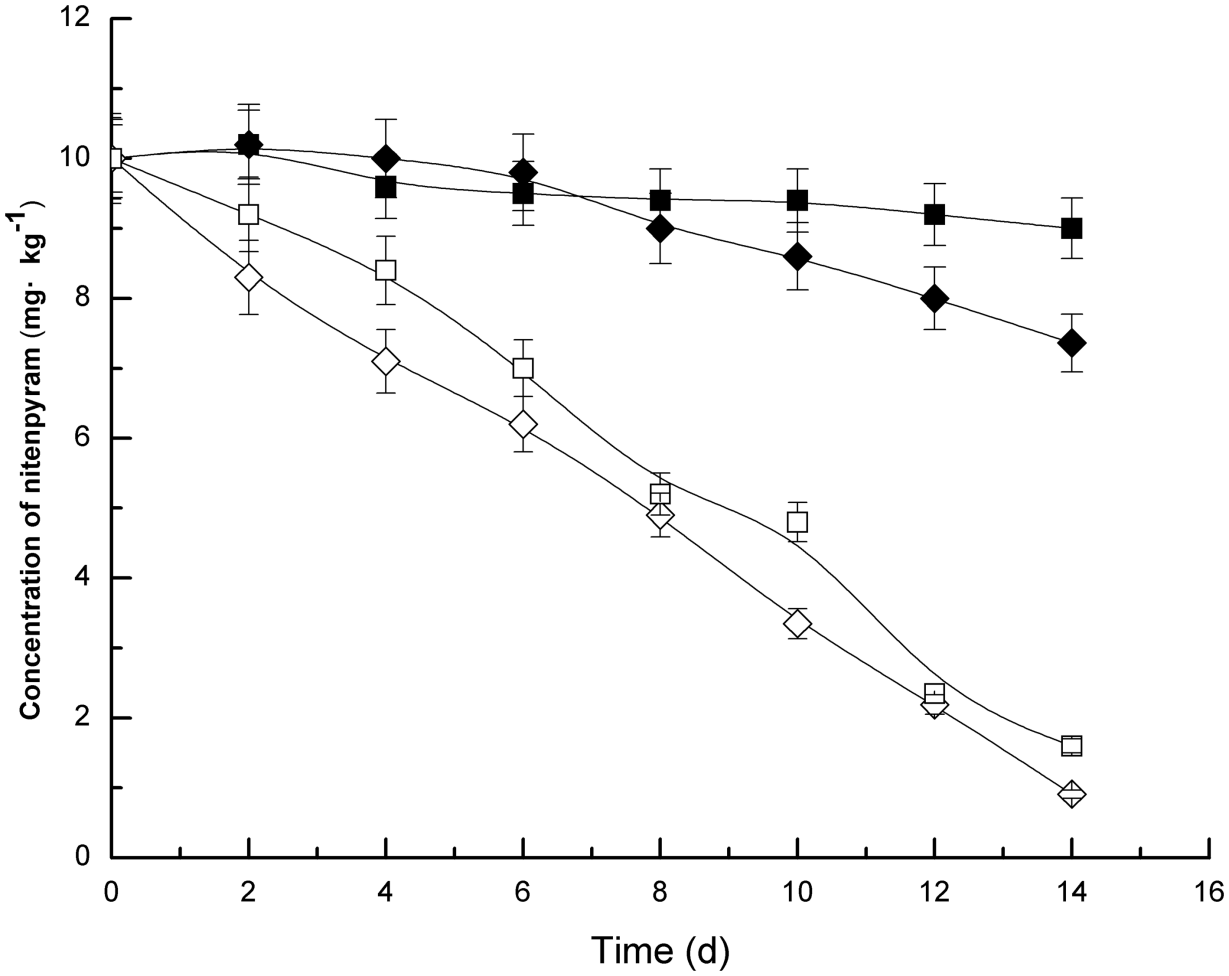
Nitenpyram degradation trend in soils. The standard error of three repetitions is expressed by the error bars. The soil uninoculated with strain DF-1 but sterilized, ■; The soil uninoculated with strain DF-1 and non-sterilized, ◆; The soil inoculated with strain DF-1 and sterilized, and □; The soil inoculated with strain DF-1 but non-sterilized, ◇.

Soil samples were inoculated with strain DF-1, which dramatically increased nitenpyram degradation in the soil. In the uninoculated sterilized soil, only approximately 10% of nitenpyram was degraded after 14 days of culture. On the contrary, the extent of nitenpyram degradation increased to 84% in strain DF-1–inoculated soil (Figure 4), indicating a substantial improvement in the efficiency of nitenpyram degradation. In fresh soil samples, after 2 weeks, 90.9% nitenpyram degradation was achieved with strain DF-1 inoculation and 26.4% was achieved without DF-1 inoculation. These results clearly imply that strain DF-1 cooperates well with indigenous soil microorganisms in degrading soil nitenpyram, thus supporting the possibility of using strain DF-1 for bioremediation of nitenpyram-polluted soil.

### 3.5. Identification of metabolites during nitenpyram degradation

**Figure 5:**
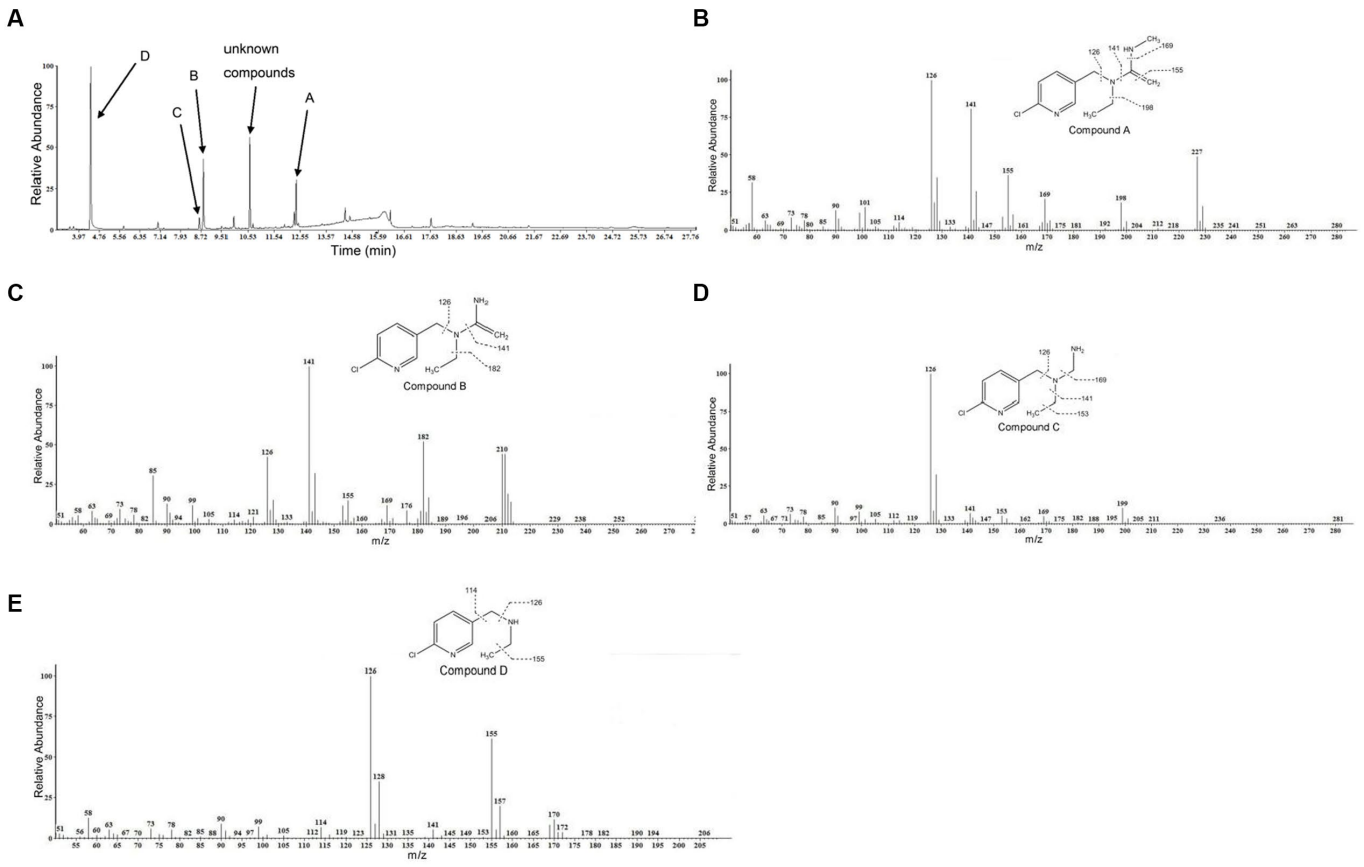
GC–MS examination of the nitenpyram intermediate forms converted by *Ochrobactrum* sp. strain DF-1: **(A)** GC profiles for the major intermediate forms A, B, C, and D. These compounds appeared at 12.403, 8.823, 8.657, and 4.426 min, respectively. **(B–E)** The typical ions of the compounds A–D through GC–MS analysis. They were recognized as N-((6-chloropyridin-3-yl)methyl)-N-ethyl-N-methylethene-1,1-diamine, N-((6-chloropyridin-3-yl)methyl)-N-ethylethene-1,1-diamine, N-((6-chloropyridin-3-yl)methyl)-N-ethylmethanediamine, and N-((6-chloropyridin-3-yl)methyl)ethanamine, respectively.

**Figure 6:**
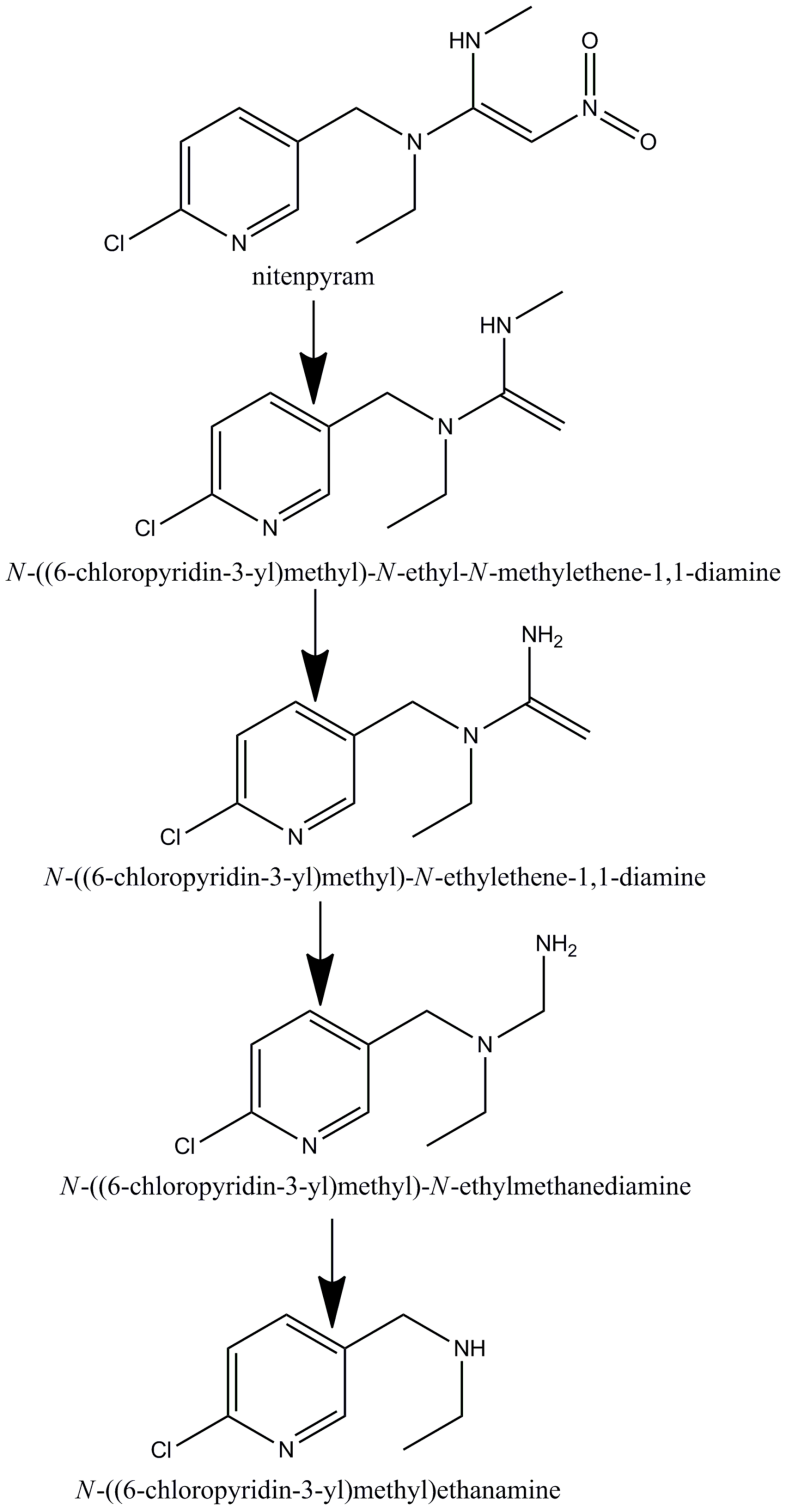
Proposed pathway for nitenpyram degradation in *Ochrobactrum* sp. DF-1.

GC–MS was used to identify metabolites produced by strain DF-1 during nitenpyram degradation. After 4 days of degradation, nitenpyram and its breakdown products were identified. The metabolite concentrations were the highest after 8 days. Then, the concentrations began to decline as the incubation duration was extended. Culture medium extracts were analyzed through GC–MS to identify nitenpyram degradation products. Four main biodegradation products were identified, with retention times (RTs) of 4.42, 8.657, 8.823, and 12.403 min, respectively, in addition to nitenpyram itself having a RT of 15.974 min (Figure 5A and Table 2). The metabolites were assigned the names A, B, C, and D based on their RTs, with nitenpyram designated as compound E. Compound A was identified as N-((6-chloropyridin-3-yl)methyl)-N-ethyl-N-methylethene-1,1-diamine (Figure 5B and Table 2), and it exhibited typical MS fragment ion peaks at m/z 126, 141, 155, 169, 198, and 227. Compound B presented a strong protonated molecular ion at 210 m/z and distinctive MS fragment ion peaks at m/z 126, 141, 155, 169, 176, 182, and 210. Therefore, it was considered as N-((6-chloropyridin-3-yl)methyl)-N-ethylethene-1,1-diamine (Figure 5C and Table 2) based on its molecular weight and distinctive fragment ion peaks. Compound C was recognized as N-((6-chloropyridin-3-yl)methyl)-N-ethylmethanediamine based on its mass spectrum and distinctive fragment ion peaks at m/z 126, 141, 153, 169, and 199 (Figure 5D and Table 2). Compound D exhibited a molecular ion at 170 m/z and was recognized as N-((6-chloropyridin-3-yl)methyl) ethanamine with typical fragment ions of 114, 126, 155, and 170 m/z for an RT of 4.426 min (Figure 5E and Table 2). A significant protonated molecular ion at m/z 374 was observed for compound E, which had an RT of 15.974 min and was comparable to the distinctive parental ion of nitenpyram at m/z (data not shown). Based on the findings of the aforementioned analysis, a catabolic pathway was proposed for nitenpyram degradation by *Ochrobactrum* sp. strain DF-1 (Figure 6).

**Table 2:** Nitenpyram metabolites identified by GC−MS.

Compound	Chemical name	Rt (min)	Characteristic ions in GC–MS (m/z)
A	N-((6-chloropyridin-3-yl)methyl)-N-ethyl-N-methylethene-1,1-diamine	12.403	126, 141, 155, 169, 198, 227
B	N-((6-chloropyridin-3-yl)methyl)-N-ethylethene-1,1-diamine	8.823	126, 141, 155, 169, 176, 182, 210
C	N-((6-chloropyridin-3-yl)methyl)-N-ethylmethanediamine	8.657	126, 141, 153, 169, 199
D	N-((6-chloropyridin-3-yl) methyl)ethanamine	4.426	114, 126, 155, 170

## 4. Discussion

Nitenpyram, a chloropyridinyl neonicotinoid, is commonly used in agricultural regions as an insecticide. Because nitenpyram causes environmental pollution, its use has been restricted in many countries owing to its suspected side-effects on non-target insect populations such as the honeybee (*Apis mellifera*). Microbial nitenpyram degradation is a potential approach for cleaning nitenpyram-contaminated sites. Microbial degradation has been proven to be a promising strategy to clean up organic environmental contaminants. Prokaryotic and eukaryotic microorganisms are capable of degrading nitenpyram (Chen et al., 2019; Wang et al., 2019; Dai et al., 2021). One white-rot fungal strain *Phanerochaete sordida* YK-624 was reported to completely degrade nitenpyram under ligninolytic conditions, while only 20% degradation was achieved under non-ligninolytic conditions. Remediation using this organism produced a novel nitenpyram metabolite, (*E*)-*N*-((6-chloropyridin-3-yl)methyl)-*N*-ethyl-*N′*-hydroxy acetimidamide (CPMHA) (Wang et al., 2019; Pang et al., 2020). Resting cells of the actinomycetes *Rhodococcus ruber* CGMCC 17550 reportedly degraded 40.60% of nitenpyram from 368.53 mmol·L^−1^ to 218.89 mmol·L^−1^ over 75 h at an optical density (OD) of 2.2 at 600 nm. Moreover, cytochrome P450 mediates the hydroxylation pathway of nitenpyram, and 1-aminobenzotriazole strongly inhibits nitenpyram degradation (Ahmad et al., 2021; Dai et al., 2021). Another fungal strain of *Aspergillus* sp., which was obtained from commercial formulation biotechnology, could degrade 92.9% nitenpyram (Chen et al., 2019; Ahmad et al., 2021). In this paper, a nitenpyram-degrading strain, DF-1, was separated from the waste water pool, and was recognized as *Ochrobactrum* sp. Other studies have indicated that *Ochrobactrum* sp. is capable of degrading vinyl chloride (Danko et al., 2004), Di-n-butyl Phthalate (Wu et al., 2010), chlorothalonil (Kim et al., 2004; Liang et al., 2010), methyl parathion (Qiu et al., 2006), tetrabromobisphenol (Zu et al., 2014), dimethyl formamide (Veeranagouda et al., 2006), marine benzo [a] pyrene (Yirui et al., 2009), acetamiprid (Wang et al., 2013), and nicotine (Yuan et al., 2007). These findings suggest that *Ochrobactrum* sp. could have a significantly potential application in the biodegradation of organic contaminants and remediation of wastewater, soil, and activated sludge. No strain from the *Ochrobactrum* genus has been documented to be competent in reducing nitenpyram. Therefore, we isolated the *Ochrobactrum* sp. DF-1 strain that utilize nitenpyram as the sole carbon or nitrogen source for growth. This bacteria metabolized 100 mg·L^−1^ nitenpyram within 10 days and efficiently degraded nitenpyram at a range of pH and temperatures. These findings suggest that strain DF-1 may be helpful for degrading nitenpyram as well as for investigating this biological method at the molecular level.

Bioremediation is a cost-effective method of converting hazardous chemicals to harmless byproducts. Numerous pesticides have been successfully removed from soil through bacterial implantation (bioaugmentation) (Jia et al., 2006; Liang et al., 2009; Li et al., 2012; Lin et al., 2023; Su et al., 2023; Zhou et al., 2023). Inoculation of *R. ruber* CGMCC 17550 enhanced nitenpyram degradation in surface water. Additionally, *R. ruber* cells immobilized by calcium-alginate remediated 87.11% of 100 mg·L^−1^ nitenpyram in 8 days (Ahmad et al., 2021; Dai et al., 2021). When strain DF-1 was added to nitenpyram-treated soil, the clearance rate was greater than that in uninoculated soils. This finding shows that strain DF-1 has the latent capability to clean nitenpyram-contaminated regions.

Studies only identify partial metabolites formed during nitenpyram degradation by a pure culture, and so, the microbial degradation pathway remained unclear. Four metabolites were identified in this study using GC–MS: N-((6-chloropyridin-3-yl)methyl) ethanamine, N-((6-chloropyridin-3-yl)methyl)-N-ethylmethanediamine, N-((6-chloropyridin-3-yl)methyl)-N-ethylethene-1,1-diamine, and N-((6-chloropyridin-3-yl)methyl)-N-ethyl-N-methylethene-1,1-diamine.

We suggested a partial transformation route for nitenpyram degradation by *Ochrobactrum* sp. DF-1 on the basis of these metabolic products (Figure 6). In the white-rot fungus *P. sordida* YK-624, the nitro group of nitenpyram was proposed to be initially reduced to form the metabolite (E)-N-((6-chloropyridin-3-yl)methyl)-N-ethyl-N0-hydroxy acetimidamide by cytochrome P450 (Wang et al., 2019). *R. ruber* CGMCC 17550 was first reported to degrade nitenpyram through a hydroxylation pathway (Dai et al., 2021). However, The bacterial strain DF-1 degraded nitenpyram through a completely new metabolic pathway, different from those of fungi and actinomycetes. The first step of this pathway is removal of a nitro group to form N-((6-chloropyridin-3-yl)methyl) ethanamine, followed by the formation of N-((6-chloropyridin-3-yl)methyl)-N-ethylmethanediamine through demethylation. Then, N-((6-chloropyridin-3-yl)methyl)-N-ethylethene-1,1-diamine is formed as a result of redox reactions. This is followed by the loss of an aminomethyl group, leading to the formation of N-((6-chloropyridin-3-yl)methyl)-N-ethyl-N-methylethene-1,1-diamine.

This experiment delivers the basis for the ongoing clarification of nitenpyram degradation by *Ochrobactrum*. Cloning the genes involved in the degradation process may offer a better understanding of nitenpyram’s biotransformation biochemistry.

## 5. Conclusion

In this study, a nitenpyram-degrading strain, DF-1, was screened from the wastewater pool of a factory. Nitenpyram degradation by this strain was simple, rapid, and highly effective. Additionally, a potential metabolic route for nitenpyram was presented. From this experiment, we can preliminarily conclude that the greater degradation rate of nitenpyram in soil supplemented with strain DF-1 indicates this bacterium’s potential for bioremediation of nitenpyram-related pollution.

The present study findings have the potential to assist the research and development of novel neonicotinoids and more generally elucidate the applicability of neonicotinoid insecticides.

## Data availability statement

The datasets presented in this study can be found in online repositories. The names of the repository/repositories and accession number (s) can be found in the article/supplementary material.

## Author contributions

GW conceived and designed the experiments. MC and YZ performed the experiments. MC and LJ wrote the manuscript. GW, MC, YZ, and LJ analyzed the data. All authors reviewed the manuscript, read, and approved the final manuscript.

## Funding

This project received a support from Provincial Natural Science Foundation of Anhui (2108085MC70), Major Program of Natural Science Research in Colleges and Universities in Anhui Province (2022AH040063), Rural Revitalization Project of Xinjiang Branch Chinese Academy of Sciences (XJFY-XCZX-20210060), the Chinese National Natural Science Foundation (32270400), and Natural Science Surplus Funding Project of Huaibei Normal University (2023ZK005).

## Conflict of interest

The authors declare that the research was conducted in the absence of any commercial or financial relationships that could be construed as a potential conflict of interest.

## Publisher’s note

All claims expressed in this article are solely those of the authors and do not necessarily represent those of their affiliated organizations, or those of the publisher, the editors and the reviewers. Any product that may be evaluated in this article, or claim that may be made by its manufacturer, is not guaranteed or endorsed by the publisher.
